# The combined survival effect of codon 72 polymorphisms and p53 somatic mutations in breast cancer depends on race and molecular subtype

**DOI:** 10.1371/journal.pone.0211734

**Published:** 2019-02-07

**Authors:** Shantel Hebert-Magee, Han Yu, Michael Behring, Trafina Jadhav, Chandrakumar Shanmugam, Andra Frost, Isam-Eldin Eltoum, Sooryanarayana Varambally, Upender Manne

**Affiliations:** Department of Pathology, University of Alabama at Birmingham, Birmingham, AL, United States of America; Wayne State University, UNITED STATES

## Abstract

**Background:**

The codon 72 polymorphism in the p53 gene relates to the risk of breast cancer (BC), but this relationship in racially diverse populations is not known. The present study examined the prognostic value of this polymorphism for African American (AA) and Caucasian (CA) BC patients separately and considered the confounding variables of molecular subtypes and somatic mutations in p53.

**Methods:**

Tissue sections of BCs from 116 AAs and 160 CAs were evaluated for p53 mutations and genotyped for the codon 72 polymorphism. The relationships of phenotypes to clinicopathologic features were determined by χ2 analyses; patient survival was estimated by Kaplan-Meier univariate and Cox regression multivariate models in a retrospective cohort study design.

**Results:**

The proportion of single nucleotide polymorphism (SNP) 72 alleles differed for races. Many cancers of AAs were Pro/Pro, but most for CAs were Arg/Arg. A higher frequency of missense p53 mutations was evident for AAs. There was an interaction between the SNP allele and p53 mutations for AA women only. The proportion of women with both the Pro/Pro allele and a p53 somatic mutation was higher for AA than CA women. The interaction between missense p53 mutations and Pro/Pro had a negative effect on survival, particularly for AAs with luminal cancers.

**Conclusions:**

For BCs, the survival effect of SNP72 combined with a p53 missense mutation is dependent on race and molecular subtype. Although such a mutation is a marker of poor prognosis, it is relevant to identify the variant Pro/Pro in the case of AAs, especially those with luminal subtypes of BC.

## Introduction

The reasons for racial differences in breast cancer (BC) incidence and mortality in the United States are not fully known. Non-Hispanic Caucasians (CAs) have a higher occurrence of BC; however, African Americans (AAs) have the poorest outcomes [1]. Although this disparity has been explained as primarily stemming from socioeconomic variations [2,3], the unequal survival among AA and CA patients is also linked to differing clinicopathologic characteristics [1,4,5]. Previous studies found that more biologically aggressive cancers contribute to the low survival rates for AA women relative to CA women [6]. Nonetheless, limited attention has been given to understanding the genetic and molecular basis for the racial discrepancy among women with BC.

For polymorphisms and somatic/missense mutations, the functional consequences of altered structures in p53 have been linked to increased risk and aggressiveness of various malignances, including BCs [7]. Although p53 is a highly conserved gene, several single nucleotide polymorphisms (SNPs) have been documented within coding and intronic regions [8–12]. The codon 72 polymorphism is a genetic variation that results in either an arginine (Arg) or a proline (Pro) residue at position 72 in the proline-rich domain (residues 64–92) of the p53 protein, resulting in a structural change in the protein [8]. For BCs, allelic differences at codon 72 are linked to altered capacity for proliferation and apoptosis [13,14]. The frequency of codon 72 alleles differs among various racial/ethnic groups [15] as well as across molecular subtypes [16]. Furthermore, in breast tissue, there is a possible connection between the p53 codon 72 polymorphism and susceptibility to somatic/missense mutations within the p53 gene [17]. Polymorphisms and somatic mutations of p53 are variables in BC progression, yet the ways in which they work together to influence the disparity in outcomes have not been identified.

In the present report, we highlight the relationship between p53 codon72 polymorphisms, racial differences, and patient survival of a cohort of AA and CA BC patients. This study brings together germline variants and somatic/missense mutations in p53 as well as molecular subtype and other clinical modifiers of race and survival of patients with BC.

## Materials and methods

### Study population

Eligible women were selected from 282 BC patients who had undergone surgical resection for a first primary BC from 1988 to 2012 at the University of Alabama at Birmingham (UAB). Formalin-fixed, paraffin-embedded (FFPE) tissue blocks from these cancers were acquired from the Anatomic Pathology Division at UAB. These histologically validated BCs and corresponding normal tissues were evaluated for the mutational status of the p53 gene, including codon 72 polymorphisms.

During the selection process, patients were excluded from the study population according to the following criteria: death within a month of surgery, incomplete follow-up, lacking subtype, unknown race, missing SNP information, or multiple malignancies. We intentionally enriched our sample for AAs and those with the triple-negative (TNBC) molecular subtype from reference population levels to allow maximum size in stratified statistical analysis. Human epidermal growth factor receptor 2 (HER2)-type tumors were not included due to a small sample size (4 patients). A waiver of consent for these studies was obtained from the Office of the Institutional Review Board for Human Use at UAB. All included patients (n = 276) had undergone surgery for primary BC at the UAB hospital. See S1 Fig in supporting file for an inclusion/exclusion flow chart.

### Pathologic features

For histologic differentiation of all cases, H&E-stained sections were reviewed individually by two pathologists (S H-M, CKS) and graded as well, moderately, or poorly differentiated (Bloom-Richardson grading system). Discordant reviews were reevaluated together to reach consensus. Well and moderately differentiated tumors were classified into grade I and grade II, respectively, and poor and undifferentiated tumors into grade III [18]. Pathologic staging was classified according to the criteria of the American Joint Commission on Cancer. The classification of BC molecular subtype was obtained by assessing estrogen receptor (ER), progesterone receptor, and human epidermal growth factor receptor 2 (HER2) by immunohistochemistry (IHC) in our diagnostic pathology laboratory, as described earlier [19]. Additionally, for this study, a small set (n = 30) of samples were validated for their ER, PR, and HER-2 status by IHC. The BC molecular subtypes determined were TNBC, luminal, and HER2-type. Since 24% of measures of Her2 were missing, to attain a viable sample size, luminal A and B types were conflated into a broad luminal subtype whenever possible. However, all cases of TNBCs were confirmed by IHC for ER, PR, HER-2 status. Three-dimensional tumor size (length, width, and depth) was taken into consideration; the largest of the three dimensions was used.

To ensure proper distinction of tumor from normal tissue, a two-step approach of cutting and staining a section from the FFPEs block was followed by macro-dissection of tumor from surrounding non-tumor tissue. Macro-dissected tumor tissues were used for DNA extraction, and, in turn, for p53 sequencing.

### Patient demographics and follow-up

Patient demographic, clinical and follow-up information were retrieved from medical records, physician charts, and pathology reports as well as from the UAB Tumor Registry. Patients were followed either by the patients’ physician or by the UAB tumor registry until their death or the date of the last documented contact within the study time frame. The Tumor Registry reassured outcome (mortality) information directly from the patients (or living relatives) and from the patients’ physicians through telephone or mail contacts. This information was again substantiated by the state death registry. Demographic data, including patient age at diagnosis, gender, self-identified race/ethnicity, date of surgery, date of the last follow-up (if alive), date of recurrence (if any), and date of death, were obtained. Menopausal status was calculated using age [20] and included the categories of premenopausal (under 45 years), perimenopausal (45–55 years), and postmenopausal (older than 55years). The Tumor Registry updated follow-up information every 6 months, and follow-up of the retrospective cohort ended in December, 2017. The laboratory investigators (HY and TJ) were blinded to the outcome information until completion of the assays.

### P53 mutational analysis and genotyping

**Fig 1:**
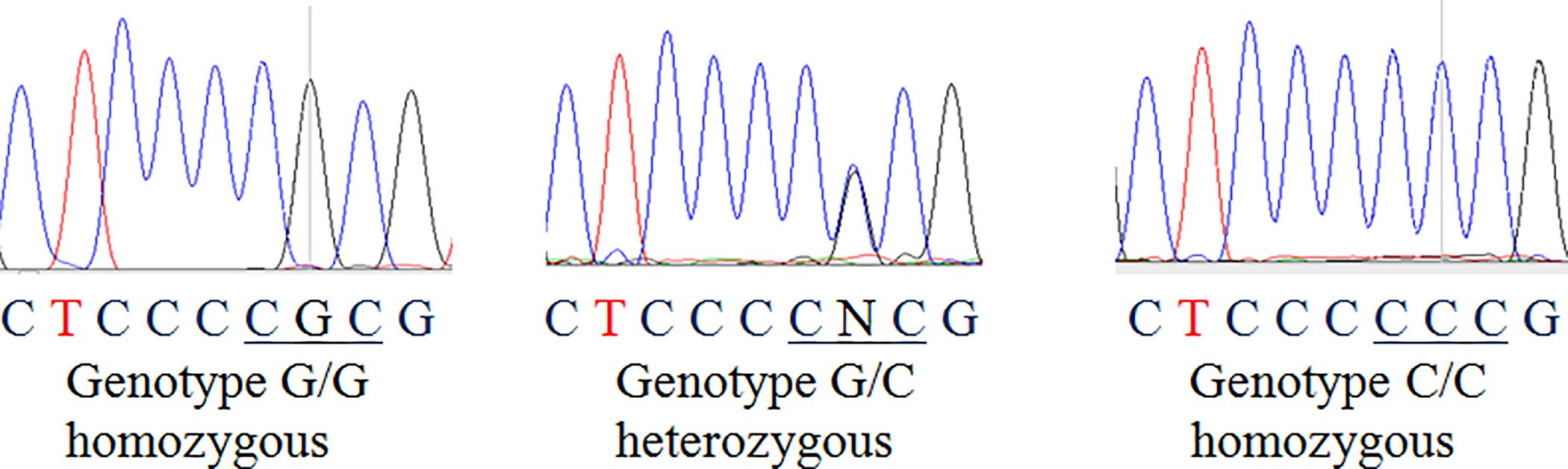
Direct sequencing analysis of DNA fragments.

DNA extraction from FFPE tissues of 276 BC patients and matching normal tissues was accomplished following a modified deparaffinization protocol [21]. The p53 gene status was determined by PCR and direct sequencing of exons 4 through 9 by use of exon-specific primers (Table 1). Exons 4, 5, 6, 7, and 8–9 of the p53 gene were amplified separately by incubating in a Thermal cycler (Bio-Rad) for 10 min at 94°C for initial denaturation, followed by 45 cycles at 94°C for 15 s, 57°C for 40 s, and 68°C for 40 s. The final extension step was 68°C for 5 min. The standard reaction mixture (25 μL) contained 10 ng of genomic DNA, 0.25 μmol/L of each primer, 0.2 μmol/L of each dNTP, 10 X PCR buffer (Invitrogen), 2 mmol/L of MgCl_2_, and 0.5 units of platinum Taq DNA polymerase (Invitrogen). Electrophoresis was performed on the final PCR products with 3% agarose gels prepared in 0.4 X Tris-borate-EDTA buffer. The purified PCR product was directly sequenced on an ABI 3100 sequencer. Sequence analysis was accomplished with Chromas Lite version 2.1.1 (Technelysium Pty, Ltd) sequencing software, which displays a representation of each nucleotide for every sequence signal (Fig 1). The sequence electrophoretograms were analyzed by manually comparing each codon with the wild type (WT) at its location to identify mutations/polymorphisms. Nucleotide differences presented within each exon sequence were validated by sequencing and analyzing the opposite strand.

**Table 1:** Oligonucleotide primer sequences for p53 gene amplification and sequencing.

**Exon**	Primers for gene amplification	Size (bp)	Primers for sequencing
**Exon-4**	S 5′TCCCCCTTGCCGTCCCAA3′	279	S 5′TCCCCCTTGCCGTCCCAA3′
	A 5′CGTGCAAGTCACAGACTT3′		A 5′CGTGCAAGTCACAGACTT3′
**Exon-5**	S 5′TTTCAACTCTGTCTCCTTCCT3′	229	S 5′CCTTCCTCTTCCTGGAGTAC3′
	A 5′GCCCCCAGCTGCTCACCATC3′		A 5′AGCTGCTCACCATCGCTATC3′
**Exon-6**	S 5′CACTGATTGCTCTTAGGTCTG3′	144	S 5′TCTTAGGTCTGGCCCCTCCT3′
	A 5′AGTTGCAAACCAGACCTCAG3′		A 5′ACCAGACCTCAGGCGGCTCA3′
**Exon-7**	S 5′GTGTTGTCTCCTAGGTTGGC3′	150	S 5′CCTAGGTTGGCTCTGACTGT3′
	A 5′TGTGCAGGGTGGCAAGTGGC3′		A 5′GGGTGGCAAGTGGCTCCTGA3′
**Exons 8–9**	S 5′CCTATCCTGAGTAGTGGTAA3′	346	S 5′TGGTAATCTACTGGGAGCAG3′
	A 5′ACTTGATAAGAGGTCCCAAG3′		A 5′CCCAAGACTTAGTACCTGAA3′

### p53 loss of heterozygosity subset analysis

The loss of heterozygosity (LOH) status of *TP53* was obtained using two microsatellite markers at *17p13*.*1* locus (TP53.PCR15, TP53.PCR18) in a subset of 94 cases randomly selected from 276 patients, as detailed in a previous publication [22]. Briefly, LOH was defined for each tumor as α = (TL1 x NL2) / (TL2 x NL1) where L is the intensity of the allele 1 or 2 in normal (N) or tumor (T) DNA. An α-score ≤ 0.5 or ≥1.5 was defined as LOH positivity. Homozygous cases were considered non-informative for LOH.

### Statistical analyses

Clinical and demographic variables were evaluated for their association to p53 polymorphisms and somatic/missense mutation variables using χ^2^ tests for categorical analysis and F-tests for continuous variables. Because a central part of this research design is oversampling of the TNBC molecular subtype; all analyses were stratified to the TNBC and luminal groups of patients. Due to the prevalence of race-dependent SNP72 alleles, this stratified analysis approach was also used for race. Probability of cancer-related death was measured using Kaplan-Meier log-rank tests for univariate relationships and Cox proportional hazard regression models for multiple variable adjusted associations. Separate Cox proportional hazard regression models were made for each race/subtype strata. The proportionality assumptions of each model were tested and found to be met, using both per-variable and global tests. We included the interaction between SNP72 and p53 somatic/missense mutation status, using all patients with WT p53 as a reference and three levels of p53 mutations by SNP72 allele. Power and sample size were calculated for subgroup analysis with the R package “powerSurvEpi” [23]. All analyses were accomplished with R statistical software version 3.4.1. Hardy-Weinberg equilibrium (HWE) tests were done using the Fisher’s exact test.

## Results

### Study cohort characteristics

This study included 276 women with BC. Information regarding patient demographics and tumor features for AA and CA patients with BC is in Table 2. AA patients tended to have a higher prevalence of TNBCs as compared with CA patients (63% vs. 43%). In contrast, CA patients presented with a higher occurrence of luminal BC (57% vs. 37%). Furthermore, AA patients were more likely to present with poorly differentiated BC with a higher Bloom-Richardson grade (grade III; 77%, χ^2^ P = 0.001). There were no significant differences by race/ethnicity with respect to age at diagnosis (χ^2^ P = 0.13), tumor stage (χ^2^ P = 0.20), tumor size (χ^2^ P = 0.31), or menopausal status (χ2 P = 0.25) (not shown), but there were significant differences with respect to the molecular subtype (χ^2^ P = 0.001) and tumor grade (χ^2^ P < 0.001).

**Table 2:** Clinicopathologic and molecular features by race.

Variable	African Americans *n* = 116	Caucasians *n* = 160	p-value
			
Mean age, years (IQR)	57 (46–68)	55 (47–63)	0.618
Tumor Stage			0.41
I	26 (22%)	50 (31%)	
II	56 (48%)	66 (41%)	
III	25 (22%)	34 (21%)	
IV	9 (8%)	10 (6%)	
Mean follow up, months (IQR)	69.4 (25.2,105.5)	93.6 (37.3,160.3)	<0.001
Molecular Subtype			0.001
Luminal	43 (37%)	92 (57.5%)	
TNBC	73 (63%)	68 (42.5%)	
Grade			<0.001
I&II	26 (23%)	68 (43%)	
III	87 (77%)	89 (57%)	
p53 status			0.002
Wild-type	75 (65%)	131 (82%)	
Mutated	41 (35%)	29 (18%)	
Codon 72			<0.001
Arg/Arg	29 (25.0%)	88 (55.0%)	
Arg/Pro	32 (28%)	30 (19%)	
Pro/Pro	55 (47%)	42 (26%)	
Interaction SNP-mutation			<0.001
Wild type	75 (65%)	131 (82%)	
Arg/Arg and p53 mutated	7 (6%)	17 (10%)	
Arg/Pro and p53 mutated	8 (7%)	6 (4%)	
Pro/Pro and p53 mutated	26 (22%)	6 (4%)	
Event measured			0.39
Alive	87 (75%)	127 (79%)	
Death from cancer	29 (25%)	33 (21%)	

Abbreviations: TNBC, triple-negative molecular subtype, n, total number of participants per group, IQR = interquartile range, mean reported

### Codon 72 polymorphism, p53 mutation, and race

Analysis of race and SNP72 revealed a higher proportion of Pro/Pro alleles in BCs of AAs than CAs with luminal or TNBC subtypes. Within TNBC tumors, SNP72 alleles showed the largest difference by race; 71% of CA TNBCs were Arg/Arg and 53% of AAs were Pro/Pro (x^2^ p-value <0.001). HWE for all participants, as well as AA and CA subgroups was rejected at <0.001 p-value. AAs had higher proportions of p53 mutations than CAs for both subtype groups, with AAs having luminal cancers showing the largest difference (x^2^ p value = 0.003). For both subtypes, the interaction of SNP72 and p53 somatic mutation differed by race. BCs of AA women with the Pro/Pro allele and any p53 mutation made up 22% of all AA BCs regardless of subtype. BCs of CA patients with any p53 mutation and the Arg/Arg allele made up 16% of all TNBC in CA women (Table 3).

**Table 3:** Association between race, molecular subtype, and clinicopathologic characteristics.

Variable	African Americans		p-value	Caucasians		p-value
	Luminal	TNBC		Luminal	TNBC	
	43 (37%)	73 (63%)		92 (58%)	68 (42%)	
Mean age, years (IQR)	59 (48–74)	55 (46–65)	0.160	55 (48–63)	55 (46–65)	0.796
Tumor Stage			>0.000			0.943
early (I & II)	33 (77%)	49 (77%)		66 (72%)	50 (73.5%)	
late (III & IV)	10 (23%)	24 (33%)		26 (28%)	18 (26.5%)	
Follow up, mean months (IQR)	63.8 (33.2–102.2)	64.9 (20.3–105.7)	0.891	85.0 (57.4–120)	72.3 (81–120)	0.053
Event (cause of death)			0.579			0.170
Alive/other	34 (79%)	53 (73%)		77 (84%)	70 (74%)	
Death from cancer	9 (21%)	20 (27%)		15 (16%)	18 (27%)	
Grade			>0.000			>0.000
I &II	18 (45%)	8 (11%)		57 (63%)	11 (17%)	
III	22 (55%)	65 (89%)		34 (37%)	55 (83%)	
p53 status			0.601			0.626
Wild-type	16 (60%)	49 (67%)		77 (84%)	54 (79%)	
Mutated	17 (40%)	24 (33%)		15 (16%)	14 (21%)	
Codon 72			0.077			0.003
Arg/Arg	10 (23%)	19 (26%)		40 (44%)	48 (71%)	
Arg/Pro	16 (40%)	15 (21%)		21 (23%)	9 (13%)	
Pro/Pro	17 (37%)	39 (53%)		31 (33%)	11 (16%)	
Interaction p53*SNP			0.451			0.167
Wild-type p53 (all SNPs)	26 (60%)	49 (67%)		77 (84%)	54 (79%)	
Arg/Arg & p53 mut	3 (7%)	4 (6%)		6 (7%)	11 (16%)	
Arg/Pro & p53 mut	5 (12%)	3 (4%)		5 (5%)	1 (2%)	
Pro/Pro & p53 mut	9 (21%)	17 (23%)		4 (4%)	2 (3%)	

Abbreviations: TNBC, triple-negative molecular subtype, n, total number of participants per group, IQR = interquartile range, mut = mutated

### p53 LOH analyses

In a subset of 94 patients that were analyzed for LOH at the *17p13*.*1* locus have shown that allelic distribution of SNP 72 was compered to overall LOH status of the p53 gene in case only cohort. LOH is commonly observed in human malignancies, including BCs, as we anticipated, our findings showed that patients with LOH (65 of 94, 70%) were associated with an increased frequency of homozygous alleles at SNP72, and deviation from HWE (Fisher exact p-value 4.1e-06). However, those cases without LOH (29 of 94, 30%) have maintained HWE (Fisher exact p-value 0.13) at this locus (Table 4).

**Table 4:** Loss of heterozygosity (LOH) and Hardy-Weinberg equilibrium.

p53 SNP72	p53 LOH markers		
	All (n = 94)	Positive (n = 65)	Negative (n = 29)
**Allele**	** **	** **	** **
G (Arg)	100	60	28
C (Pro)	88	70	30
**Genotype**			
GG (Arg/Arg)	32	23	9
GC (Arg/Pro)	24	14	10
CC (Pro/Pro)	38	28	10
**HWE Exact test**	1.70E-06	4.10E-06	0.1341

### Survival analyses

**Fig 2:**
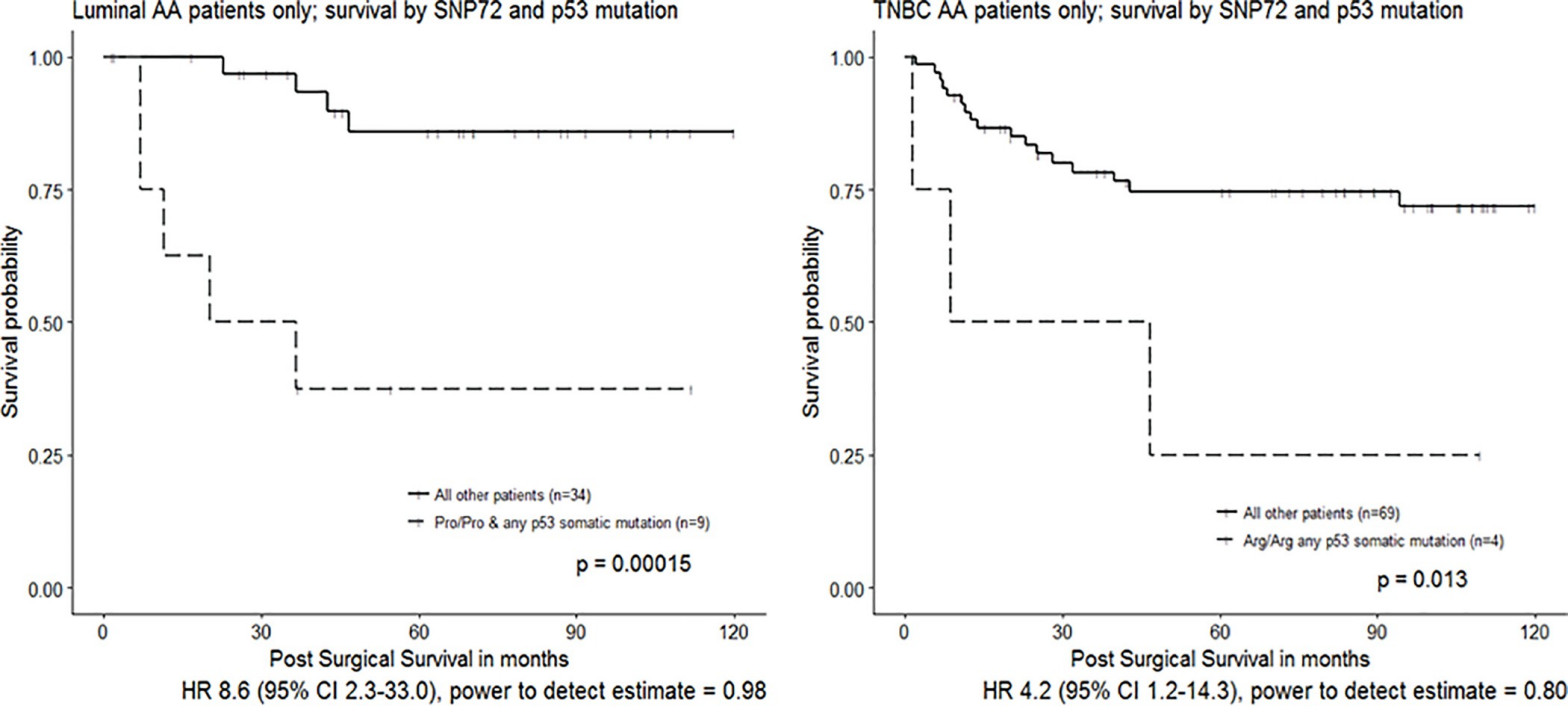
Kaplan-Meier log-rank 10-year survival curves for African American patients, split by molecular subtype (luminal and TNBC). Interaction effect for SNP72 allele and somatic p53 mutation.

Unadjusted Kaplan-Meier analyses found that p53 mutations and SNP72 have a race-dependent influence on patient survival. For AAs, both SNP72 and p53 mutation status were associated with increased probability of death from cancer; for CAs, there was no association (S2 Fig). When both p53 somatic mutation and SNP72 allele were evaluated together, there was a combined negative influence on survival that was dependent on both race and molecular subtype. After stratifying each cancer subtype by race (Table 5), in a pairwise analysis of p53 and SNP interaction, for CAs there was no significant combined effect of SNP and somatic mutation on survival. For AAs, the effect of SNP-by-somatic mutation was confounded by molecular subtype. For AA women with luminal tumors, those having Pro/Pro alleles and any p53 mutation had 8.5 times higher hazard of death from cancer than other AA women with luminal cancers (Fig 2). For TNBCs among AAs only, the Arg/Arg genotype paired with any somatic p53 mutation had 4.2 times greater hazard of death from cancer than other AA women with TNBCs.

**Table 5:** Univariate hazard of death from cancer by subtype and race.

Variable	African Americans		Caucasians	
	Luminal Subtype	TNBC Subtype	Luminal Subtype	TNBC Subtype
	43 (37%)	73 (63%)	92 (58%)	68 (42%)
Age, years (IQR)	1.01 (0.96–1.05)	1.02 (0.99–1.06)	1.01 (0.97–1.07)	1.01 (0.98–1.04)
Tumor Stage				
early (I & II)	ref	ref	ref	ref
late (III & IV)	7.85 (2.03–30.28)	7.24 (2.82–18.60)	13.54 (4.26–43.01)	7.35 (2.83–19.10)
Grade				
I &II	ref	ref	ref	ref
III	0.56 (0.13–2.34)	1.08 (0.25–4.65)	1.63 (0.57–4.70)	0.52 (0.19–1.47)
p53 status				
Wild-Type	ref	ref	ref	ref
Mutated	7.41 (1.54–35.74)	1.37 (0.56–3.35)	0.73 (0.16–3.25)	1.76 (0.63–4.95)
Codon 72				
Arg/Arg	4.27 (0.39–47.19)	2.99 (0.81–11.07)	0.47 (0.13–1.76)	—
Arg/Pro	ref	ref	ref	ref
Pro/Pro	9.39 (1.12–78.48)	0.98 (0.26–3.69)	0.74 (0.23–2.43)	—
Interaction p53*SNP				
Wild-type (all SNPs)	ref	ref	ref	ref
Arg/Arg & p53 mutated	7.77 (0.70–86.09)	4.14 (1.17–14.69)	—	1.88 (0.61–5.77)
Arg/Pro & p53 mutated	2.29 (0.21–25.29)	1.26 (0.16–9.75)	2.52 (0.57–11.20)	—
Pro/Pro & p53 mutated	13.59 (2.59–71.18)	0.92 (0.30–2.86)	—	2.48 (0.32–19.06)

Abbreviations: TNBC, triple-negative molecular subtype, n, total number of participants per group, IQR = interquartile range, mean reported

Adjusted analysis of codon 72 SNP and p53 somatic/missense mutation interaction was restricted by small sample sizes, particularly for the luminal AA subset. As determined with an age- and stage- adjusted Cox model for TNBC of AAs, any Arg allele and p53 mutation had a 3.16 times increased hazard of death from cancer (95% CI 0.94–10.61) (Table 6). With a stage-adjusted Cox model for luminal-only AA women, the estimate of increased hazard for those with any Pro allele and p53 somatic/missense mutation was HR 7.11, 95% CI 1.01–50.19. The models had power to detect the hazard estimate at 0.86 and 0.87, respectively.

**Table 6:** Cox regression analysis to determine prognostic significance of p53 somatic mutation and codon 72 phenotypes.

Prognostic variables			Indicator of poor prognosis	Hazard ratio (95% confidence intervals)		p-value
						
**African American patients**						
						
**Luminal subtype**						
Tumor Stage						
Late (III&IV) vs. Early (I&II)			late stage	2.97	(0.60–14.82)	0.185
SNP72 p53 interaction						
Arg/Arg or Arg/Pro + p53 mutation vs. wild type			proline allele	2.96	(0.40–21.80)	0.290
Pro/Pro + p53 mutation vs. wild type			proline allele	7.11	(1.01–50.19)	0.049
						
**TNBC subtype**						
Age, years			increased age	1.04	(0.99–1.07)	0.069
Tumor Stage						
Late (III&IV) vs. Early (I&II)			late stage	10.50	(3.82–28.80)	<0.000
SNP72 p53 interaction						
Arg/Arg or Arg/Pro + p53 mutation vs. wild type			arginine allele	3.16	(0.94–10.61)	0.062
Pro/Pro + p53 mutation vs. wild type			arginine allele	0.65	(0.20–2.09)	0.473

SNP 72 and p53 mutation interaction terms were combined to account for the effect of single alleles

## Discussion

Through this study, we demonstrated relationships between race, molecular subtype, SNP72, and somatic/missense mutations of p53 and survival for women with BC. In general, BCs of AA Pro/Pro patients were more susceptible to also exhibiting somatic/missense mutations in p53. This interaction between the germline p53 genotype and somatic/missense mutation was a predictor of survival for AAs based on molecular subtype. For cancers of AA TNBC women, the SNP 72 Arg/Arg variant along with p53 somatic mutation conveyed the poorest survival. However, for AA patients with luminal BCs, the SNP72 Pro/Pro variant and p53 somatic/missense mutation showed the worst survival.

Although most polymorphisms are not harmful, some have the capacity to alter gene expression or coded protein functions. These functional polymorphisms, including the codon 72 polymorphisms of p53, have different incidence among races and contribute to vulnerability and severity of diseases. The prevalence of polymorphisms of codon72 (rs1042522) varies depending on population ethnicity. For healthy CAs of European descent, most were Arg/Arg (~55%) followed by heterozygous Arg/Pro phenotypes. For healthy AAs, most were Pro/Pro and heterozygous Pro/Arg (~40% each) [24,25]. Our results suggest that, although Arg alleles are more prevalent for CAs, they confer no increase in either incidence of p53 mutation or hazard of cancer-related death. However, for AAs, the abundance of Pro alleles was associated with an increase in p53 somatic/missense mutations and those mutations had a negative effect on survival that was evident only for women with luminal tumors.

In the current study population, made up of case-only women with BC, the trend of high frequencies of Arg/Arg alleles in CAs and Pro/Pro in AAs was consistent with previous studies. This is also indicative of LOH which has been associated with cancer in general [26], and specifically within the p53 gene in BCs [27]. In a subset analysis, the effect of LOH in p53 upon SNP72, we found that, for patients without LOH, HWE was maintained, while patients with LOH had strongly rejected HWE as anticipated. These findings, and the known associations of LOH in p53 in breast cancer, indicate that HWE for SNP72 was confounded by malignancy-related LOH in this study, rather than bias (Table 4).

A prior study suggested an interaction between somatic mutant forms of p53 and SNP72 Arg/Arg, which, in BCs, conveys prognostic results different from WT p53 [28]. Indeed, in the present study, Arg/Arg patients with p53 somatic/missense mutations had poor survival. However, this relationship was dependent on race and TNBC subtype. The only previous research on the topic of molecular subtype and SNP72 found a positive, but non-significant, association between Arg alleles and ER-positive tumors of European women [29]. The present analysis confirms that CA women had more of the Arg/Arg genotype than AAs regardless of subtype, with the most marked difference for TNBC tumors.

Previous research has shown that both the TNBC molecular subtype and p53 mutations are higher for AAs and that they have an influence on shorter time to recurrence for AAs versus CAs [30]. In the present study, we observed a similar combined effect of race, subtype, and p53 somatic/missense mutation incidence and added a discovery of the importance of underlying p53 polymorphisms. We found that the survival effect by race was altered by subtype when SNP72 status was combined with p53 somatic/missense mutation as an interaction term.

Missense mutations contribute to more than 85% of p53 somatic mutations. Moreover, for several cancers, including BCs, overexpression of mutant p53 (nuclear accumulation of p53, detected by immunohistochemistry) correlates with more advanced tumor development and worse patient survival [31,32]. Missense mutations can lead to single amino acid substitutions that alter the primary structure of the p53 protein and lead to loss of its function [33]. Among AAs who exhibited SNP 72 Pro/Pro and any p53 somatic mutation, there was a higher proportion of missense point mutations in comparison to CAs with the same Pro/Pro allele (21/26, for AAs and 4/6, for CAs, respectively). Furthermore, AA women with luminal tumors exhibited a higher proportion of Pro/Pro SNP72 and missense point mutations of p53 as compared to CAs. Likewise, this group of AA women with Pro/Pro and mutated p53 in their BCs had a higher proportion of disruptive p53 somatic mutations (Table 7). These findings propose that reasons for racial disparity in outcomes lie not only in frequency of molecular subtype and p53 mutation but also are reliant upon how germline variants of SNP72 work together with both to effect survival.

**Table 7:** Descriptive features of SNP72 and p53-mutated cancers by race and luminal subtype.

Race	Subtype	Age	Cancer death	SNP72	Stage	Codon p53	Consequence of mutation	Amino acid change
AA	Luminal	61	yes	Pro/Pro	late	65	nondisruptive	Arg > Lys
AA	Luminal	93	yes	Pro/Pro	early	191	disruptive	Pro—> Ser
AA	Luminal	62	no	Pro/Pro	early	213	disruptive	Arg>Stop
AA	Luminal	57	no	Pro/Pro	early	184	disruptive	Asp—>Asn
AA	Luminal	62	yes	Pro/Pro	late	157	nondisruptive	Val—>Ala
AA	Luminal	86	no	Pro/Pro	late	55	nondisruptive	Thr > Ser
AA	Luminal	29	yes	Pro/Pro	late	136	disruptive	Gln-> His
AA	Luminal	58	no	Pro/Pro	early	184	disruptive	Asp—>Asn
AA	Luminal	36	yes	Pro/Pro	late	204	disruptive	Glu—>Stop
CA	Luminal	84	no	Pro/Pro	early	69	nondisruptive	Ala > Asp
CA	Luminal	50	no	Pro/Pro	early	213	disruptive	Arg>Stop
CA	Luminal	66	no	Pro/Pro	early	237	nondisruptive	Met—>Ile
CA	Luminal	67	no	Pro/Pro	early	213	disruptive	Arg>Stop

A limitation of this study was oversampling by race and molecular subtype, sample size, and power for interaction of SNP72 and p53 somatic mutation. The small size for interaction analysis meant that adjusted Cox models were underpowered, and that stratified univariate analyses were the best option. In an effort to address this, we included power calculations for HR estimates in all relevant covariate stratified Kaplan-Meier results.

In conclusion, the increased occurrence of p53 mutations cancers of AA women was associated with Pro/Pro phenotypes, and AA patients having both p53 somatic/missense mutations and the Pro/Pro allele had significantly shorter survival, particularly with luminal subtypes. Although these correlations need to be validated in large prospective studies, the findings suggest that, in combination with other indicators of disease development, analysis of the codon 72 polymorphism of the p53 gene together with somatic mutations can aid in understanding racial differences in progression of BCs, in identifying aggressive forms, and in designing optimal therapies.

## Supporting information

S1 Fig Inclusion flow diagram. (DOCX)

S2 Fig Kaplan-Meier log-rank 10 year survival curves by SNP72 and p53 mutational status; A) African American survival by SNP72 allele; B) Caucasian survival by SNP72 allele; C) African American survival by p53 somatic mutation; D) Caucasian survival by p53 somatic mutation.
(DOCX)

S1 Table Association between p53 codon 72 phenotypes and clinicopathologic characteristics. (DOCX)

S2 Table Clinicopathologic and molecular features; univariate hazard of death from cancer by race. (DOCX)
