# Supplementary material for: The combined survival effect of codon 72 polymorphisms and p53 somatic mutations in breast cancer depends on race and molecular subtype
Source: PLoS One. 2019 Feb 7;14(2):e0211734. doi: 10.1371/journal.pone.0211734 (PMC6366783; doi:10.1371/journal.pone.0211734)
Supplement: S1 Fig — (DOCX) [file pone.0211734.s001.docx]

**Supplemental Figures and Tables**

**S1 Fig.** Inclusion flow diagram
