# Supplementary material for: The combined survival effect of codon 72 polymorphisms and p53 somatic mutations in breast cancer depends on race and molecular subtype
Source: PLoS One. 2019 Feb 7;14(2):e0211734. doi: 10.1371/journal.pone.0211734 (PMC6366783; doi:10.1371/journal.pone.0211734)

**S2 Fig.** Kaplan-Meier log-rank 10 year survival curves by SNP72 and p53 mutational status; A) African American survival by SNP72 allele; B) Caucasian survival by SNP72 allele; C) African American survival by p53 somatic mutation; D) Caucasian survival by p53 somatic mutation


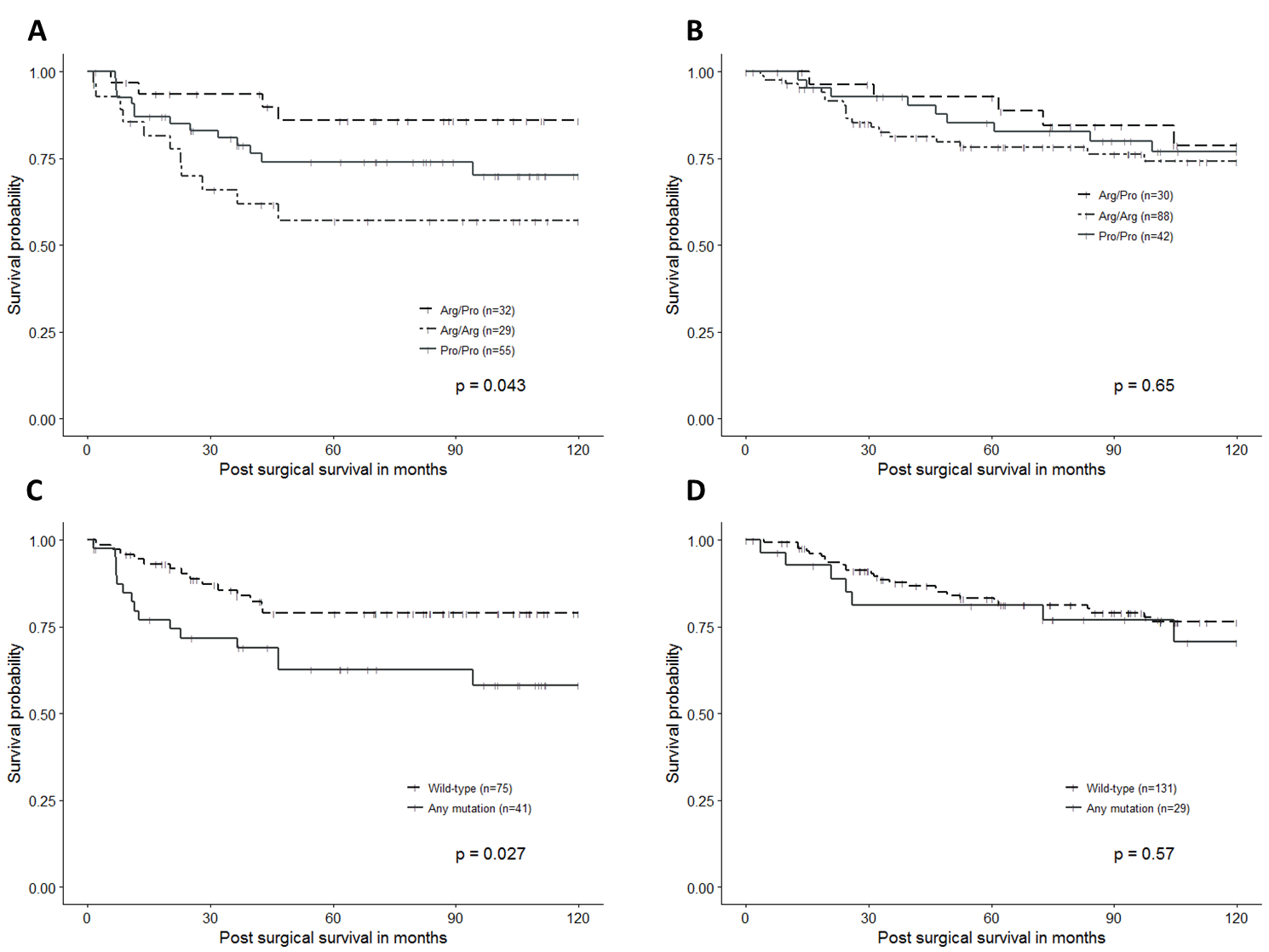

Supplement: S2 Fig — Kaplan-Meier log-rank 10 year survival curves by SNP72 and p53 mutational status; A) African American survival by SNP72 allele; B) Caucasian survival by SNP72 allele; C) African American survival by p53 somatic mutation; D) Caucasian survival by p53 somatic mutation. (DOCX) [file pone.0211734.s002.docx]
