# Supplementary material for: The combined survival effect of codon 72 polymorphisms and p53 somatic mutations in breast cancer depends on race and molecular subtype
Source: PLoS One. 2019 Feb 7;14(2):e0211734. doi: 10.1371/journal.pone.0211734 (PMC6366783; doi:10.1371/journal.pone.0211734)
Supplement: S1 Table — (DOCX) [file pone.0211734.s003.docx]

|  | **S1 Table.** Association between p53 codon 72 phenotypes and clinicopathologic characteristics | | | | | | | | | | | |  |  |
| --- | --- | --- | --- | --- | --- | --- | --- | --- | --- | --- | --- | --- | --- | --- |
|  | **Variable** |  | **African Americans, *n*=116** | | |  | **p-value** |  | **Caucasians, *n*= 160** | | |  | **p-value** |  |
|  |  |  | **Arg/Arg** | **Arg/Pro** | **Pro/Pro** |  |  |  | **Arg/Arg** | **Arg/Pro** | **Pro/Pro** |  |  |  |
|  |  |  | **29 (25.0%)** | **32 (27.6%)** | **55 (47.4%)** |  |  |  | **88 (55.0%)** | **30 (18.8%)** | **42 (26.2%)** |  |  |  |
|  |  |  |  |  |  |  |  |  |  |  |  |  |  |  |
|  | Mean age, years (IQR) | | 53 (45,63) | 62 (46,74) | 57 (47,64) |  | 0.194 |  | 56 (48-64) | 56 (45-63) | 55 (47-63) |  | 0.751 |  |
|  | Tumor Stage |  |  |  |  |  |  |  |  |  |  |  | 0.288 |  |
|  | I |  | 5 (17.2%) | 13 (40.6%) | 8 (14.5%) |  |  |  | 25 (28.4%) | 10 (33.3%) | 15 (35.7%) |  |  |  |
|  | II |  | 14 (48.3%) | 14 (43.8%) | 28 (50.9%) |  |  |  | 37 (42%) | 10 (33.3%) | 19 (45.2%) |  |  |  |
|  | III |  | 7 (24.1%) | 2 (6.2%) | 16 (29.1%) |  |  |  | 18 (20.5%) | 10 (33.3%) | 6 (14.3%) |  |  |  |
|  | IV |  | 3 (10.3%) | 3 (9.4%) | 3 (5.5%) |  |  |  | 8 (9.1%) | 0 (0%) | 2 (4.8%) |  |  |  |
|  | Follow up, mean months (IQR) |  | 42.7  (12.8-98.2) | 82.7  (43.4-111.14) | 70.5  (25.5-104.5) |  | 0.101 |  | 73.9  (28.1-130.5) | 98.2  (61.8-162) | 127.6  (73.3-209.8) |  | 0.003 |  |
|  | Molecular subtype | | |  |  |  | 0.077 |  |  |  |  |  | 0.003 |  |
|  | Luminal |  | 10 (34.5%) | 17 (53.1%) | 16 (29.1%) |  |  |  | 40 (45.5%) | 21 (70%) | 31 (73.8%) |  |  |  |
|  | TNBC |  | 19 (65.5%) | 15 (46.9%) | 39 (70.9%) |  |  |  | 48 (54.5%) | 9 (30%) | 11 (26.2%) |  |  |  |
|  | Grade* |  |  |  |  |  | 0.609 |  |  |  |  |  | 0.836 |  |
|  | I &II |  | 8 (27.6%) | 9 (25.8%) | 12 (18.9%) |  |  |  | 48 (41.2%) | 14 (46.7%) | 19 (45.2%) |  |  |  |
|  | III |  | 21 (72.4%) | 23 (74.2%) | 43 (81.1%) |  |  |  | 50 (58.8%) | 16 (53.3%) | 23 (54.8%) |  |  |  |
|  | p53 status |  |  |  |  |  | 0.038 |  |  |  |  |  | 0.751 |  |
|  | Wild-type |  | 22 (75.9%) | 24 (75%) | 29 (52.7%) |  |  |  | 71 (80.7%) | 24 (80%) | 38 (85.7%) |  |  |  |
|  | Mutated |  | 7 (24.1%) | 8 (25%) | 26 (47.3%) |  |  |  | 17 (19.3%) | 6 (20%) | 6 (14.3%) |  |  |  |
|  |  |  |  |  |  |  |  |  |  |  |  |  |  |  |
|  | Abbreviations: TNBC, triple-negative molecular subtype, *n*, total number of participants per group, IQR = interquartile range  * Information on tumor grade was not available for 3 AAs and 3 CAs | | | | | | | | | | | | |  |
|  |  |  |  |  |  |  |  |  |  |  |  |  |  |  |
|  |  |  |  |  |  |  |  |  |  |  |  |  |  |  |
