# Supplementary material for: The combined survival effect of codon 72 polymorphisms and p53 somatic mutations in breast cancer depends on race and molecular subtype
Source: PLoS One. 2019 Feb 7;14(2):e0211734. doi: 10.1371/journal.pone.0211734 (PMC6366783; doi:10.1371/journal.pone.0211734)
Supplement: S2 Table — (DOCX) [file pone.0211734.s004.docx]

|  | **S2 Table.** Clinicopathologic and molecular features; univariate hazard of death from cancer by race | | | | | |
| --- | --- | --- | --- | --- | --- | --- |
|  | **Variable** |  | **African Americans, n=116** |  | **Caucasians, n= 160** |  |
|  |  |  |  |  |  |  |
|  | Age at diagnosis |  | 1.02 (0.99-1.04) |  | 1.01 (0.98-1.04) |  |
|  | Tumor Stage |  |  |  |  |  |
|  | Early (stage I &II) |  | ref |  | ref |  |
|  | Late ( stage III&IV) |  | 7.53 (3.49-16.23) |  | 9.17 (4.41-19.06) |  |
|  | Molecular subtype |  |  |  |  |  |
|  | Luminal |  | ref |  | ref |  |
|  | TNBC |  | 1.36 (0.62-3.00) |  | 1.85 (0.93-3.67) |  |
|  | Grade* |  |  |  |  |  |
|  | I&II |  | ref |  | ref |  |
|  | III |  | 0.96 (0.41-2.26) |  | 1.34 (0.66-2.71) |  |
|  | p53 status |  |  |  |  |  |
|  | Wild-Type |  | ref |  | ref |  |
|  | Mutated |  | 2.23 (1.07-4.62) |  | 1.27 (0.55-2.94) |  |
|  | Codon 72 |  |  |  |  |  |
|  | Arg/Arg |  | 3.92 (1.25-12.32) |  | 1.52 (0.57-4.07) |  |
|  | Arg/Pro |  | ref |  | ref |  |
|  | Pro/Pro |  | 2.27 (0.75-6.91) |  | 1.19 (0.40-3.56) |  |
|  | Interaction SNP-mutation |  |  |  |  |  |
|  | Wild type |  | ref |  | ref |  |
|  | Arg/Arg and p53 mutated |  | 4.60 (1.51-14.00) |  | 1.37 (0.48-3.93) |  |
|  | Arg/Pro and p53 mutated |  | 1.20 (0.27-5.27) |  | 1.63 (0.39-6.89) |  |
|  | Pro/Pro and p53 mutated |  | 2.15 (0.93-4.96) |  | 0.74 (0.10-5.45) |  |
|  |  |  |  |  |  |  |
|  | Abbreviations: TNBC, triple-negative molecular subtype, *n*, total number of participants per group * Information on tumor grade was not available for 3 AAs and 3 CAs | | | | | |
|  | * Information on tumor grade was not available for 3 AAs and 3 CAs | | | | | |
|  |  |  |  |  |  |  |
